# Effect of Zr Addition on the Microstructure and Mechanical Properties of CoCrFeNiMn High-Entropy Alloy Synthesized by Spark Plasma Sintering

**DOI:** 10.3390/e20110810

**Published:** 2018-10-23

**Authors:** Hongling Zhang, Lei Zhang, Xinyu Liu, Qiang Chen, Yi Xu

**Affiliations:** 1 School of materials Science & Engineering, Southwest Jiaotong University, Chengdu 610031, China; 2 Chengdu Advanced Metal Materials Industry Technology Research Institute Co., Ltd, Chengdu 610031, China

**Keywords:** high-entropy alloy, mechanical alloying, spark plasma sintering, nanoprecipitates, mechanical properties

## Abstract

As a classic high-entropy alloy system, CoCrFeNiMn is widely investigated. In the present work, we used ZrH_2_ powders and atomized CoCrFeNiMn powders as raw materials to prepare CoCrFeNiMnZr*_x_* (*x* = 0, 0.2, 0.5, 0.8, 1.0) alloys by mechanical alloying (MA), followed by spark plasma sintering (SPS). During the MA process, a small amount of Zr (*x* ≤ 0.5) can be completely dissolved into CoCrFeNiMn matrix, when the Zr content is above 0.5, the ZrH_2_ is excessive. After SPS, CoCrFeNiMn alloy is still as single face-centered cubic (FCC) solid solution, and CoCrFeNiMnZr*_x_* (*x* ≥ 0.2) alloys have two distinct microstructural domains, one is a single FCC phase without Zr, the other is a Zr-rich microstructure composed of FCC phase, B2 phase, Zr_2_Ni_7_, and σ phase. The multi-phase microstructures can be attributed to the large lattice strain and negative enthalpy of mixing, caused by the addition of Zr. It is worth noting that two types of nanoprecipitates (body-centered cubic (BCC) phase and Zr_2_Ni_7_) are precipitated in the Zr-rich region. These can significantly increase the yield strength of the alloys.

## 1. Introduction

During the past few years, high-entropy alloys (HEAs), a type of multi-principal-element alloy, have drawn widespread attention form worldwide material scientists [1,2,3]. Compared to conventional alloys, which are composed of one or two major elements, HEAs usually contain 5–13 principal elements, and the content of each component is between 5 and 35 at.% [4]. Due to the high mixing entropy in the multi-component alloy systems, HEAs generally form a simple face-centered cubic (FCC) or body-centered cubic (BCC) structure solid solution and exhibit many novel properties, for example, superb mechanical properties [5,6], outstanding resistances to wear [7], oxidation [8] and corrosion [9], as well as a good temperature stability [10]. Therefore, the concept of HEAs provides a new approach to design alloys with excellent properties to meet different environmental requirements. 

Casting [11] and powder metallurgy [12] are two common methods for preparing HEAs. For casting, there are two kinds of defects which are the vaporization of low melting-point elements and the elements segregation in the ingot [13]. For powder metallurgy, the HEAs can be prepared by MA and then consolidated. The HEAs powders prepared by MA are uniform and fine, and even form nanocrystalline. Hence a rapid sintering is needed to maintain this microstructure [14]. SPS is a new field-assisted sintering technique which can effectively suppress the grain coarsening by rapid heating and efficient densification in a few minutes [15,16,17]. Compared with MA, gas atomization can produce more homogeneous powders. The preparation of HEAs powders by gas atomization is a rapid solidification process [18], in which grain growth and element segregation are inhibited, and the high cooling rate even leads to the formation of an amorphous phase [14]. 

In the previous studies, two classical HEAs, CoCrFeNiMn with a single FCC phase and AlCoCrFeNi with a single BCC phase, have been extensively studied. Among these studies, there are lots of experiments that have explored the influence of alloying elements such as Mo [5], Nb [6], Ti [19], Si [20], and Zr [21] on the microstructure and properties of AlCoCrFeNi. The addition of these alloying elements leads to the formation of intermetallic compounds and significantly improves the yield strength of AlCoCrFeNi. For example, after the addition of Zr, Laves phase appears in the BCC-phase matrix, the yield strength of AlCoCrFeNi increases from 1320 MPa to 1560 MPa, and the plastic strain increases from 22.5% to 29.5%. It is feasible to change the phase composition and improve the mechanical properties of AlCoCrFeNi by adding an alloying element. However, there are little similar data on the CoCrFeNiMn alloy [22,23]. In the present work, the CoCrFeNiMn alloy was selected as the matrix and the effect of Zr addition was investigated. There are several reasons for choosing Zr as an alloying element. Firstly, both Zr and Ti are located in the same family in the periodic table of elements, so they have some similar physico-chemical properties. In previous research, the addition of Ti can result in precipitates and improve the superplasticity of CoCrFeNiMn [23]. Secondly, the large atomic size difference and negative enthalpies of mixing [24] between Zr and other constituent elements can produce a strong lattice distortion and even change the phase composition, which may improve the mechanical properties of the CoCrFeMnNi. 

In this paper, we focused on the novel high-entropy alloy system of CoCrFeNiMnZr*_x_* (*x* = 0, 0.2, 0.5, 0.8, 1.0). First of all, CoCrFeNiMn metallic powders were prepared by gas atomization, and then, Zr derived from the in-situ dehydrogenation of ZrH_2_ powders was solid-dissolved into CoCrFeNiMn by MA. The CoCrFeNiMnZr*_x_* alloy powders were subsequently sintered by SPS. The alloying behavior, microstructures, and mechanical properties of both powders and sintered alloys were investigated.

## 2. Experimental

Equimolar ratio CoCrFeNiMn powders were prepared by gas atomization with high purity Ar. The atomization pressure was 4 MPa. Then, using CoCrFeNiMn powders and ZrH_2_ powders as raw materials, the CoCrFeNiMnZr*_x_* (*x* values in molar ratio, *x* = 0, 0.2, 0.5, 0.8 and 1.0, denoted by Zr_0_, Zr_0.2_, Zr_0.5_, Zr_0.8_, and Zr_1.0_, respectively) alloys in nominal compositions were prepared by high-energy ball milling (referred as MA) and SPS. The reaction (ZrH_2_ = Zr(s) + H_2_(g)) occurs during MA and SPS. In this experiment, the mixed powders and zirconia balls (5 mm and 10 mm in diameter with the mass ration of 1:1) were put into stainless-steel vials at a mass ratio of 10:1, then mounted on a planetary ball miller (QM-S3P4, Nanjing NanDa Instrument Plant, Nanjing, China) and milled at a rate of 300 rpm under the protection of argon gas. All the mixed powders were milled for 30 h and stopped for 10 min every 20 min to prevent the powders from overheating. Beforehand, in order to study the alloying behavior, mixed Zr_0.5_ alloy powders were milled for 50 h, and a small amount of powder was taken every 10 h for X-ray diffraction tests. Finally, the gas-atomized CoCrFeNiMn powders without ball milling (Zr_0_w) and the alloy powders with 30 h of ball milling were sintered by SPS (Dr. Sinter-3.20 MKII, SCM, Japan) at 900 °C for 8 min under a uniaxial pressure of 40 MPa with a vacuum of 1 × 10^–3^ Pa. The sintered columnar samples have a diameter of 30 mm and a height of 10 mm.

The oxygen content of the CoCrFeNiMn powder was analyzed by the fusion method on an O/N analyzer (736 series, LECO, Saint Joseph, MI, USA). X-ray diffraction with Cu Kα radiation (XRD, XPertPowder, PANalytical, Almelo, Netherlands) was used to analyze the phase composition of the alloy powder and bulk at a speed of 4°/min, and the 2 thetas ranging from 20° to 100°. The PDF-2 2004 database was used for the phase assignment. The morphology and chemical composition were characterized by scanning electron microscopy (SEM, Quanta, FEI, Hillsboro, TX, USA) equipped with energy dispersive spectrometry (EDS, Inca X-Max, Oxford instruments, Oxford, UK) operating at 20 kV. Cuboidal specimens with a side length of 10 mm and a height of 5 mm were cut from sintered samples and then ground and polished for SEM and EDS analyses. The crystal structure was identified by transmission electron microscopy (TEM, JEM2100F, JEOL, Tokyo, Japan) operating at 200 kV. The disc-shaped TEM samples with a diameter of 3 mm were electropolished (TenuPol-5, Struers, Ballerup, Denmark) in an electrolyte composed of 20 vol.% perchloric acid and 80 vol.% methanol at –15 °C, and the applied voltage and current were 60 mV and 80 mA, respectively. Archimedes’ method was used to measure the density of samples. Cylindrical specimens (7 mm in height and 3.5 mm in diameter) were used for the compressive tests (810 series, MTS, Minneapolis, MN, USA) at room temperature. Samples of each composition were tested for 3 times.

## 3. Results and Discussion

### 3.1. Phase and Microstructure of the Gas Atomized HEA Powders

**Figure 1:**
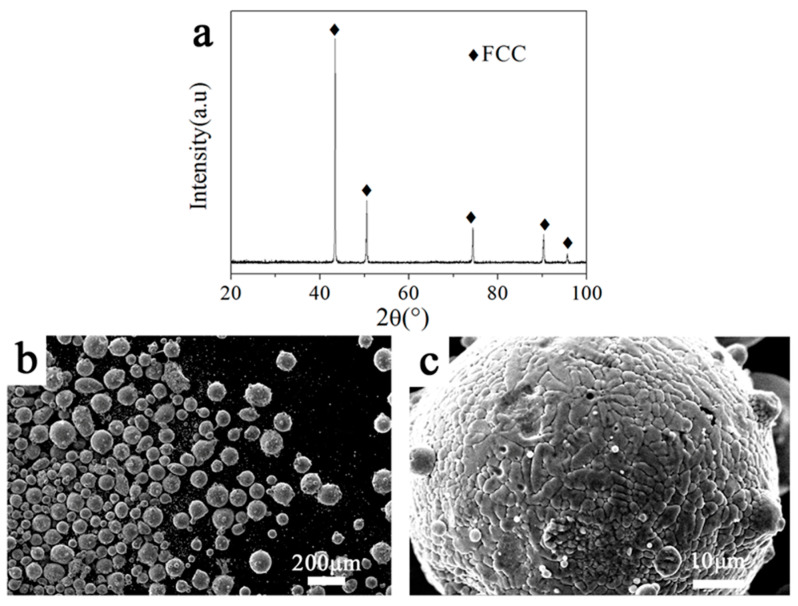
The X-ray diffraction (XRD) pattern (**a**), morphology (**b**) and microstructure (**c**) of the gas-atomized Zr_0_ HEA powders.

The chemical analysis of the atomized HEA powder is shown in Table 1. As can be seen, the composition of the CoCrFeNiMn powder prepared by gas atomization was consistent with the nominal composition. The low oxygen content of the powder means that very little oxidation occurred during the atomization. The XRD pattern indicates the formation of the CoCrFeNiMn HEA powders with a single FCC phase (Figure 1a). The morphology and microstructure of the gas-atomized powders are depicted in Figure 1b,c. The atomized powders are spherical or quasi-spherical in diameter, ranging from 5 to 150 μm, and a small amount of fine powders adhere to the surface of large powders to form satellite structures. During the process of gas atomization, solidified fine droplets can easily adhere to the surface of large droplets that remain molten. The zoom-in image indicates that the microstructure consists of cellular and dendritic structures at a submicron scale.

### 3.2. Microstructure and Phase Evolution during MA

**Figure 2:**
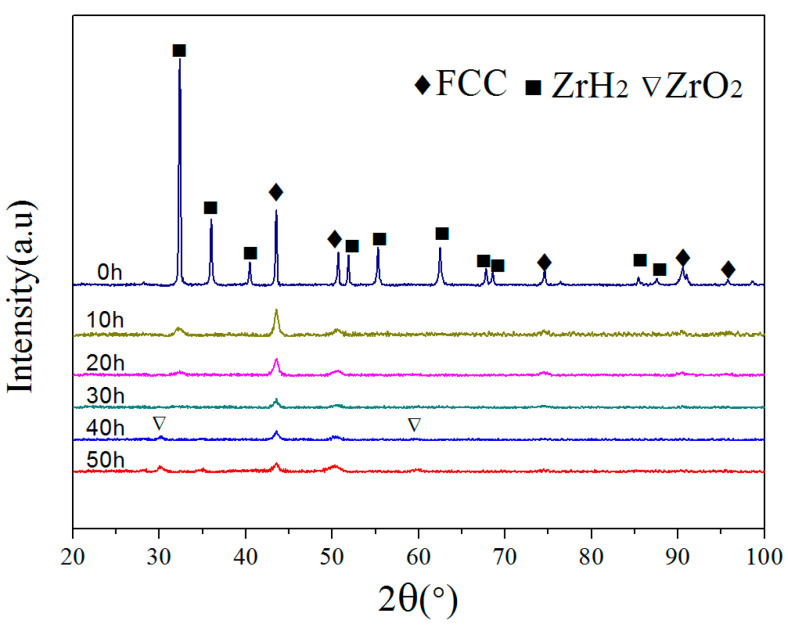
The XRD patterns of the Zr_0.5_ alloy powders with different milling times.

To investigate the alloying behavior in the MA process, X-ray diffraction (XRD) was performed on the milled Zr_0.5_ powders at 10 h intervals until the milling time reached 50 h (Figure 2). It can be seen that the XRD pattern of primary blending powders includes diffraction peaks of CoCrFeNiMn alloy with FCC structure and ZrH_2_ (PDF 073-2076). After 10 h of ball milling, the diffraction peaks of ZrH_2_ could still be detected, but their intensity was significantly reduced. This indicates that ZrH_2_ decomposed and the Zr dissolved into the matrix. After 20 h of ball milling, some diffraction peaks of ZrH_2_ disappeared due to the alloying. As the milling time increasing to 30 h, all the diffraction peaks of ZrH_2_ became absent and the XRD pattern became similar to that of Figure 1, which suggests that the Zr was fully dissolved into the FCC matrix to form a supersaturated solid solution. Additionally, as the ball milling time was extended, the diffraction peaks appeared to broaden. Throughout the milling process, the lattice strain increase and grain refinement are the main reasons for the peak broadening and intensity reduction [25]. On the other hand, ZrO_2_ diffraction peaks were observed after 40 h of ball milling, and the intensity of the ZrO_2_ diffraction peaks increase with the extending of ball milling time. The raw materials are usually contaminated by milling media in the process of ball milling [26,27]. 

Table 2 shows the average crystal size and lattice strain of the Zr_0.5_ alloy powders with different milling times that have been calculated using the Williamson–Hall equation method. As the milling time was extended from 10 h to 50 h, the crystal size decreased from 14.8 nm to 8.1 nm, and the lattice strain increased from 0.237% to 0.746%. Thus, the high lattice strain and the formation of nanocrystalline are the main reasons for the mentioned diffraction peaks broadening and the diffraction intensity reduction.

**Figure 3:**
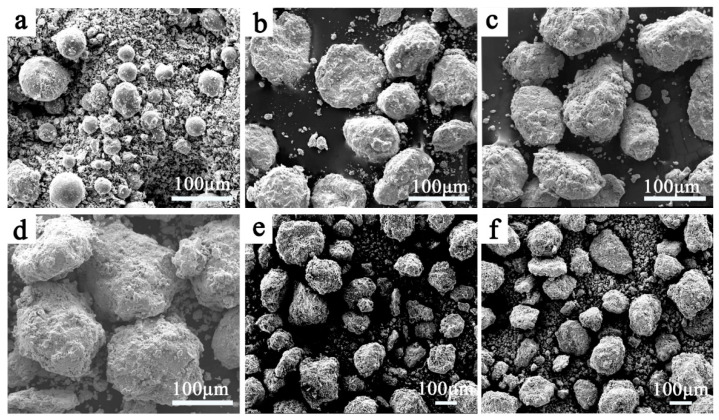
The scanning electron microscopy (SEM) images of the Zr_0.5_ alloy powders with different milling times: (**a**) 0 h; (**b**) 10 h; (**c**) 20 h; (**d**) 30 h; (**e**) 40 h; (**f**) 50 h.

Figure 3 shows the morphologies of the Zr_0.5_ alloy powders that have undergone different milling times. The initial powder is a mixture of spherical atomized CoCrFeNiMn powders and irregularly shaped ZrH_2_ powders, and the size of the ZrH_2_ powders are smaller than the CoCrFeNiMn powders (Figure 3a). The difference in size between the two powders will affect the alloying process. After 10 h of ball milling, the relatively large-sized CoCrFeNiMn powders became elliptical and fine sheet powders were deposited on their surfaces. This indicates that the alloy powders were deformed, broken, and welded during ball milling and, at the same time, the fine Zr (or ZrH_2_) powders and broken CoCrFeNiMn powders were agglomerated on the surface of the large powder particles. As the ball milling time extended, the CoCrFeNiMn powders were wrapped by Zr-rich alloy powders and the outer layer of the CoCrFeNiMn powders was alloyed with Zr under the impact of the high-energy small balls. Meanwhile, the cycle of crushing and agglomeration of the outer layer continued, which causes the powders to gradually refine and promotes the alloying and diffusion among the different alloy elements [28,29]. After 30 h of ball milling, the particle size hardly changed, which means that the crushing and agglomeration reached a dynamic balance in the MA process.

**Figure 4:**
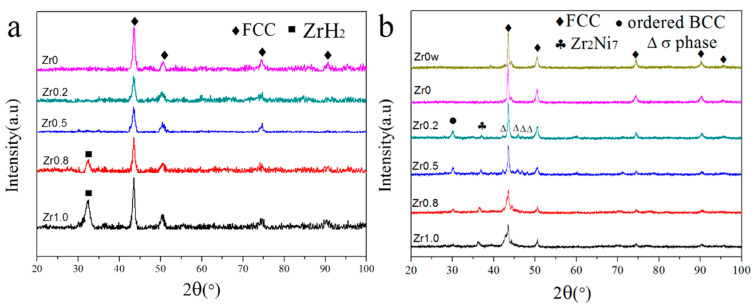
The XRD patterns of HEAs: (**a**) CoCrFeNiMnZr*_x_* (*x* = 0, 0.2, 0.5, 0.8, 1.0) alloy powders after milling for 30 h, (**b**) sintered CoCrFeNiMnZr*_x_* (*x* = 0w, 0, 0.2, 0.5, 0.8, 1.0) HEAs.

Figure 4a displays the XRD patterns of HEA powders with different contents of Zr after ball milling of 30 h. Compared with Figure 2, no extra peaks can be found in the XRD patterns of the Zr_0_, Zr_0.2_, and Zr_0.5_ alloys, which suggests that the ZrH_2_ was completely decomposed and the Zr was effectively dissolved into the CoCrFeNiMn HEA matrix when the Zr content is not higher than 0.5. Nevertheless, the diffraction peak of ZrH_2_ can be found in the XRD patterns when the Zr content is up to 0.8 and 1.0, and the intensity of the peak increases with the increase of the ZrH_2_ content.

### 3.3. Phase Evolution and Microstructure after SPS

XRD patterns of the sintered CoCrFeNiMnZr*_x_* (*x* = 0w, 0, 0.2, 0.5, 0.8, 1.0) alloys are shown in Figure 4b. Obviously, the ZrH_2_ diffraction peaks in the Zr_0.8_ and Zr_1.0_ powders disappear in the sintered samples. This suggests that the excessive ZrH_2_ was decomposed and that the Zr remainder was fully alloyed during SPS. The FCC phase is still the dominant phase, meanwhile, some weak diffraction peaks which are identified as Zr_2_Ni_7_ (PDF 071-0543), σ phase, and ordered BCC (B2) phase appear. Compared with the XRD patterns of the powders after ball milling, the broad diffraction peaks became narrow due to internal energy releases during the SPS process. 

The Zr_0_w and Zr_0_ alloys have the same diffraction peaks and consist of an FCC phase, indicating that the MA and SPS processes do not alter the FCC phase of the alloys. The diffraction peaks of the Zr_2_Ni_7_, the B2 phase, and the σ phase can be found in the Zr_0.2_, Zr_0.5_, Zr_0.8_, and Zr_1.0_ alloys. The σ phase was widely reported in previous research [30,31] and it can be identified as the NiCoCr (PDF 021-1271) σ phase, which has a tetragonal structure (a = 8.85 Å and c = 4.59 Å) in the present experiment. The B2 phase has been often found in HEAs containing CoCrFeNi [4,21,32]. These indicate that during the SPS process, some of the alloy atoms diffuse under thermal activation conditions to form ordered solid solutions and intermetallic compounds. 

With the increase of the Zr content, the diffraction intensity of the B2 phase decreases while the diffraction intensity of the Zr_2_Ni_7_ compound increases. This indicates that when the Zr content is low, the free energy is reduced by the formation of the ordered phase. However, as the Zr content increases, the formation of the Zr_2_Ni_7_ is more effective to reduce the free energy, and the formation of the B2 phase is suppressed. On the other hand, the diffraction peaks of the σ phase almost disappear in the Zr_1.0_ alloy, which also means that the formation of the Zr_2_Ni_7_ suppresses the formation of the σ phase. These can be attributed to the lattice strain and a lager negative enthalpy of mixing, caused by the addition of Zr.

**Figure 5:**
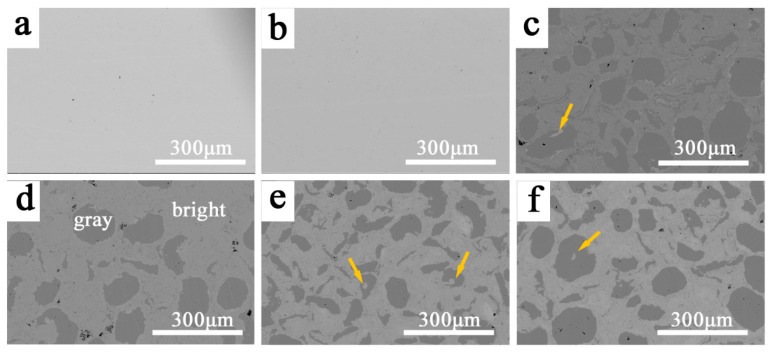
The SEM (operating at back-scattered electron (BSE) mode) images of the CoCrFeNiMnZr*_x_* alloys synthesized by mechanical alloying (MA) and spark plasma sintering (SPS). (**a**) *x* = 0w; (**b**) *x* = 0; (**c**) *x* = 0.2; (**d**) *x* = 0.5; (**e**) *x* = 0.8; (**f**) *x* = 1.0. Any Zr-rich particles were wrapped by a plastic face-centered cubic (FCC) phase and indicated by yellow arrows.

Figure 5 displays the SEM (operating at back-scattered electron (BSE) mode) images of the CoCrFeNiMnZr*_x_* alloys synthesized by MA and SPS. As can be seen from Figure 5a,b, both the Zr_0_w and Zr_0_ alloys exhibit a single phase feature, which is consistent with the XRD results. After the addition of Zr, there are two distinct microstructural domains: the bright and gray regions (Figure 5c–f).

**Figure 6:**
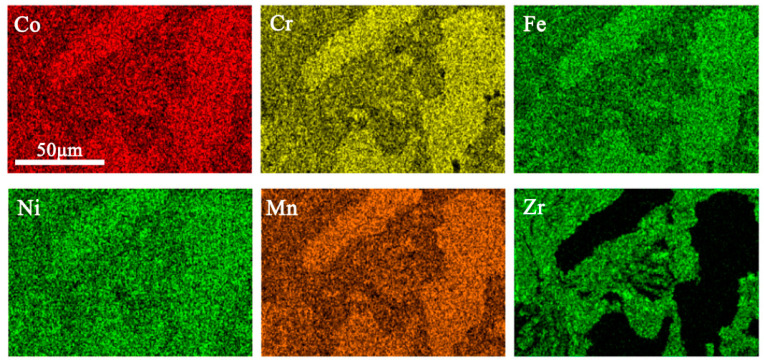
The energy dispersive spectrometry (EDS) mappings of the Zr_0.2_ alloy synthesized by MA and SPS.

EDS was performed to investigate the two regions using the Zr_0.2_ alloy and the results are shown in Figure 6 and Table 3. On the one hand, the bright region contains a higher amount of Zr than the nominal composition whereas Zr is barely found in the gray regions. On the other hand, the content of Co, Cr, Fe, Ni, and Mn are relatively lower in the bright region, and they are almost equal in the atomic ratio in both regions. Obviously, some CoCrFeNiMn HEA particles are not completely broken by the high-energy ball milling due to their high toughness [14,33], and Zr is not uniformly distributed in the matrix. The gray regions can be identified as a pure CoCrFeNiMn FCC phase and the bright region consists of FCC phase, B2 phase, Zr_2_Ni_7_, and σ phase. For the gray region, the shapes present are ovals and strips, which were inherited from the MA process. Furthermore, the hard and brittle Zr-rich particles were wrapped by a plastic FCC phase during the ball milling process, a few small bright microstructures are indicated by yellow arrows (Figure 5). Table 4 shows the density of the sintered CoCrFeNiMnZr*_x_* alloys. It can be seen that the addition of a relatively low-density Zr element reduces the density of the alloy system (the density of Co, Cr, Fe, Ni, Mn, and Zr are 8.9 g·cm^−3^, 7.19 g·cm^−3^, 7.87 g·cm^−3^, 8.9 g·cm^−3^, 7.44 g·cm^−3^, and 6.49 g·cm^−3^, respectively).

**Figure 7:**
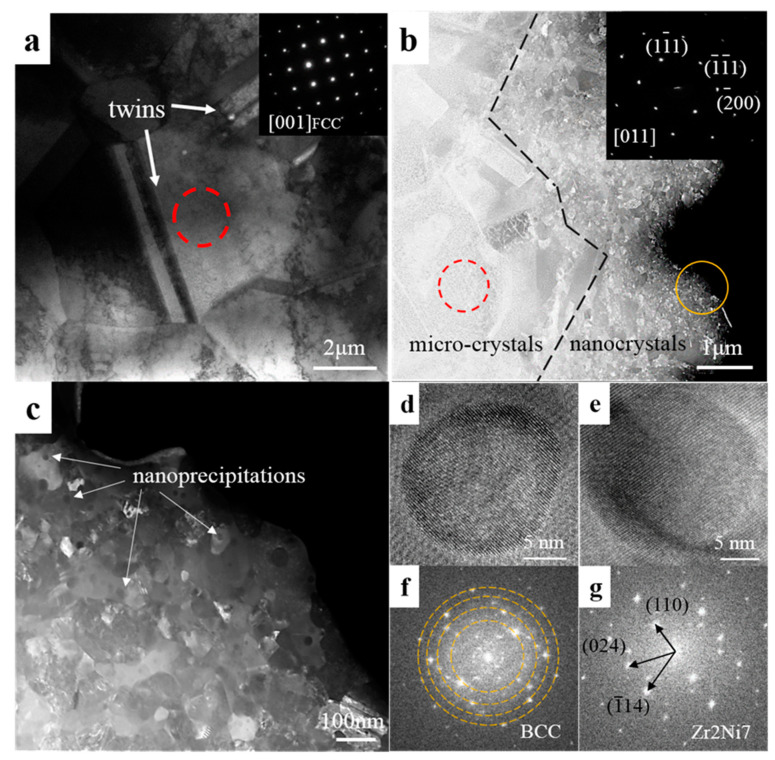
The transmission electron microscopy (TEM) images of sintered (**a**) Zr_0_ and (**b**) Zr_0.2_ HEAs. The insets in (**a**,**b**) are the selected area electron diffraction (SAED) patterns of the red circle marking area. The dashed line in (**b**) distinguishes the two regions with different grain sizes: the micro-crystals on the left side of the dashed line and the nanocrystals on the right side of the dashed line; (**c**) is the magnifying image of the yellow circle marking area in (**b**); (**d**,**e**) are the high-resolution TEM (HRTEM) of nanoprecipitation; (**f**,**g**) are the fast Fourier transformations (FFT) of (**d**,**e**), respectively.

Figure 7a shows the grain structure of Zr_0_ and the selected area electron diffraction (SAED) pattern is related to the red circle marking area. It indicates that the sintered Zr_0_ alloy has a micron grain size and a single FCC structure which is consistent with XRD results. Meanwhile, some twins are found. After the Zr addition, there are two obviously different areas distinguished by grain size (Figure 7b). The SAED pattern of red circle marking area demonstrates that the micro-crystal has an FCC structure. EDS results (not shown here) revealed that these nanocrystals are Zr-rich while the micro-crystals do not contain Zr. This confirms the SEM results: the Zr and the outer layer of the atomized powder are alloyed during the ball milling process, and as time passes, the fine Zr-rich powders agglomerated on the outer layer is continuously refined. Furthermore, many spherical nanoprecipitates are found in the nanocrystal area (Figure 7c). Figure 7d–g show the high-resolution TEM (HRTEM) images with corresponding fast Fourier transformations (FFT) of nanoprecipitates. These indicate that there are two kinds of nanoprecipitates which are BCC structure precipitates and Zr_2_Ni_7_ precipitates. In the previous studies [3,34], nanoprecipitates have been considered to be beneficial to the mechanical properties of the material due to the ability of pinning dislocations.

### 3.4. Mechanical Properties

**Figure 8:**
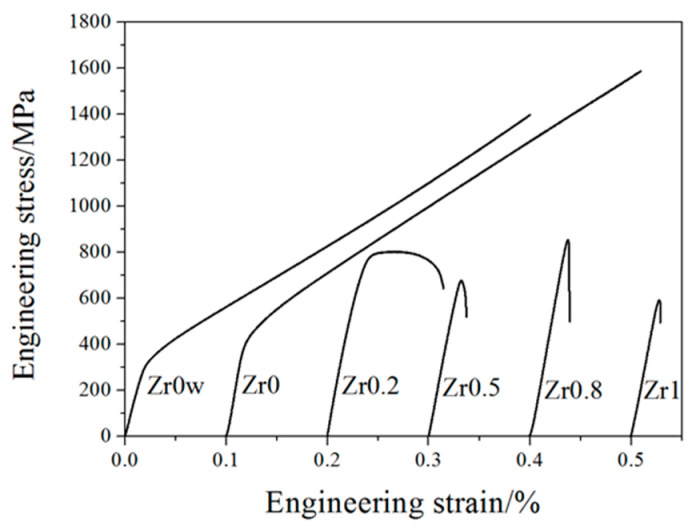
The compressive engineering stress–strain curves of the CoCrFeNiMnZr*_x_* alloys.

Figure 8 shows the compressive stress-strain curves of the CoCrFeNiMnZr*_x_* alloys at room temperature. The values of yield strength (YS) σ*_y_*, compressive strength (CS) σ_max_, and plastic strain limit (PS) ε*_p_* of alloys are listed in Table 5. The maximum yield strength reaches 820 MPa when the Zr content is up to 0.8, but the compressive strength of the Zr*_x_* (*x* = 0.2, 0.5, 0.8, 1.0) alloys are lower than that of alloys without Zr. Because of the addition of Zr, the yield strength evidently improved while the ductility declined simultaneously. Zr_0_ and Zr_0_w alloys, exhibit excellent plasticity, and both of the two alloys are not broken in the pressure range of the compressor. The yield strength of Zr_0_ is higher than that of Zr_0_w, which suggests that MA can improve the strength of the alloy by refining the grain [14]. Furthermore, the yield strength of the CoCrFeNiMnZr*_x_* (*x* = 0.2, 0.5, 0.8, 1.0) alloys are significantly higher than that of the Zr_0_ alloy, but the plasticity strongly reduced at the same time. This can be attributed to solid solution strengthening and second phase strengthening caused by the addition of Zr, as well as fine grain strengthening obtained by mechanical ball milling.

**Figure 9:**
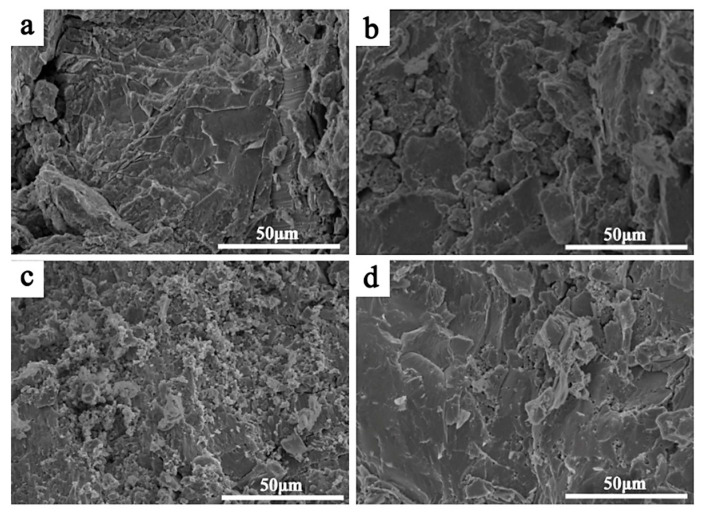
SEM micrographs of the fracture surface of CoCrFeNiMnZr*_x_* alloys. (**a**) *x* = 0.2; (**b**) *x* = 0.5; (**c**) *x* = 0.8; (**d**) *x* = 1.0.

The fractographs of the CoCrFeNiMnZr*_x_* (*x* = 0.2, 0.5, 0.8, 1.0) alloys after compressive tests are depicted in Figure 9. The fracture surface of the Zr_0.2_ alloy presents a step-like pattern with quasi-cleavage characteristics. A small radiation zone appears on the right side of Figure 9a, indicating the localized ductile fracture. Complete cleavage fracture patterns can be seen in Figure 9b–d, which thus deteriorates the alloy plasticity. For the Zr_0.5_ alloy, the fracture surface consists of many small cleavage planes, with both inter-granular fractures and trans-granular fractures having occurred. Large bright and flat cleavage planes appear in the fractures of Zr_0.5_ and Zr_1.0_ alloys, which is in accord with the totally brittle fracture of the alloys. The above results indicate that the CoCrFeNiMnZr*_x_* (*x* = 0.2, 0.5, 0.8, 1.0) alloys exhibit brittle fractures, which can be attributed to a large lattice distortion and the brittle intermetallic compounds present in the Zr-rich region.

## 4. Conclusions

The alloying behavior, microstructures, and mechanical properties of both powders and bulk CoCrFeNiMnZr*_x_* alloys were investigated. The main conclusions of the present work are given below:

(1) Metastable single FCC phase CoCrFeNiMnZr*_x_* alloy powders were prepared after 30 h of ball milling, which means that MA could enhance the solid solubility of large size elements in the matrix. However, toughness powder is difficult to break during ball milling, which would affect the alloying process and lead to the element segregation. Toughness powders can retain their original properties during ball milling, which means that MA can be used to prepare HEAs composites.

(2) The phase composition of HEAs can be changed by adjusting the content of the alloying element. After SPS, CoCrFeNiMn maintains a single FCC phase. However, two distinct microstructural domains, one composed of a micro-sized CoCrFeNiMn alloy without Zr and the other one composed of Zr-rich multi-phase microstructures consisting of a nano-sized FCC phase, B2 phase, Zr_2_Ni_7_, and σ phase, appear in the CoCrFeNiMnZr*_x_* (*x* ≥ 0.2) alloys. When the elements with different physicochemical properties are dissolved into the HEA matrix, the local structural stability will be destroyed, and the local atomic arrangement will change from disorder to order. 

(3) The addition of Zr significantly increases the yield strength of the CoCrFeNiMn alloy. The Zr_0.8_ alloy exhibits the highest yield strength up to 820 MPa which is almost twice as much as the Zr_0_ alloy (420 MPa). The high yield strength makes it a promising candidate for precision equipment manufacturing. 

## Figures and Tables

**Table 1:** The chemical compositions of the gas atomized CoCrFeNiMn high-entropy alloy (HEA) powder.

Elements	Co	Cr	Fe	Ni	Mn	O
at.%	20.12	18.85	19.45	20.05	19.71	0.0365

**Table 2:** The crystal size and lattice strain of the Zr_0.5_ HEA powders with different milling times.

Milling Time (h)	Crystal Size (nm)	Lattice Strain (%)
10	14.8	0.237
20	13.4	0.385
30	12.5	0.473
40	11.2	0.564
50	8.1	0.746

**Table 3:** The chemical compositions of the Zr_0.2_ alloy synthesized by MA and SPS.

Elements	Co	Cr	Fe	Ni	Mn	Zr
Gray region (at.%)	19.2	21.0	20.0	18.9	20.9	0
Bright Region (at.%)	14.9	16.3	15.0	14.9	16.1	22.8

**Table 4:** The density of the CoCrFeNiMnZr*_x_* alloys synthesized by MA and SPS.

Samples	Zr_0_w	Zr_0_	Zr_0.2_	Zr_0.5_	Zr_0.8_	Zr_1.0_
Density (g/cm^3^)	7.91 ± 0.03	7.92 ± 0.02	6.86 ± 0.04	6.87±0.05	6.91 ± 0.03	6.93 ± 0.02

**Table 5:** The mechanical properties of the CoCrFeNiMnZr*_x_* alloys.

*x*	YS σ*_y_* (MPa)	CS σ_max_ (MPa)	PS ε*_p_* (%)
0w	350	>1400	>40
0	420	>1600	>40
0.2	800	880	12
0.5	800	810	4
0.8	820	850	3
1.0	600	600	2.5
